# Improvement Using Planomics Features on Prediction and Classification of Patient-Specific Quality Assurance Using Head and Neck Volumetric Modulated Arc Therapy Plan

**DOI:** 10.3389/fnins.2021.744296

**Published:** 2021-10-01

**Authors:** Bing Li, Junying Chen, Wei Guo, Ronghu Mao, Xiaoli Zheng, Xiuyan Cheng, Tiantian Cui, Zhaoyang Lou, Ting Wang, Dingjie Li, Hongyan Tao, Hongchang Lei, Hong Ge

**Affiliations:** ^1^Department of Radiation Oncology, Affiliated Cancer Hospital of Zhengzhou University, Henan Cancer Hospital, Zhengzhou, China; ^2^State Key Laboratory of Oncology in South China, Collaborative Innovation Center for Cancer Medicine, Sun Yat-sen University Cancer Center, Guangzhou, China; ^3^Department of Planning and Finance, Affiliated Cancer Hospital of Zhengzhou University, Henan Cancer Hospital, Zhengzhou, China

**Keywords:** VMAT, H&N, quality assurance, radiotherapy, machine learning

## Abstract

**Purpose:** This study aimed to evaluate the utility of a new plan feature (planomics feature) for predicting the results of patient-specific quality assurance using the head and neck (H&N) volumetric modulated arc therapy (VMAT) plan.

**Methods:** One hundred and thirty-one H&N VMAT plans in our institution from 2019 to 2021 were retrospectively collected. Dosimetric verification for all plans was carried out using the portal dosimetry system integrated into the Eclipse treatment planning system based on the electronic portal imaging devices. Gamma passing rates (GPR) were analyzed using three gamma indices of 3%/3 mm, 3%/2 mm, and 2%/2 mm with a 10% dose threshold. Forty-eight conventional features affecting the dose delivery accuracy were used in the study, and 2,476 planomics features were extracted based on the radiotherapy plan file. Three prediction and classification models using conventional features (CF), planomics features (PF), and hybrid features (HF) combining two sets of features were constructed by the gradient boosting regressor (GBR) and Ridge classifier for each GPR of 3%/3 mm, 3%/2 mm, and 2%/2 mm, respectively. The absolute prediction error (APE) and the area under the curve (AUC) were adopted for assessing the performance of prediction and classification models.

**Results:** In the GPR prediction, the average APE of the models using CF, PF, and HF was 1.3 ± 1.2%/3.6 ± 3.0%, 1.7 ± 1.5%/3.8 ± 3.5%, and 1.1 ± 1.0%/4.1 ± 3.1% for 2%/2 mm; 0.7 ± 0.6%/2.0 ± 2.0%, 1.0±1.1%/2.2 ± 1.8%, and 0.6 ± 0.6%/2.2 ± 1.9% for 3%/2 mm; and 0.4 ± 0.3%/1.2 ± 1.2%, 0.4±0.5%/1.3 ± 1.0%, and 0.3±0.3%/1.2 ± 1.1% for 3%/3 mm, respectively. In the regression prediction, three models give a similar modeling performance for predicting the GPR. The classification results were 0.67 ± 0.03/0.66 ± 0.07, 0.77 ± 0.03/0.73 ± 0.06, and 0.78 ± 0.02/0.75 ± 0.04 for 3%/3 mm, respectively. For 3%/2 mm, the AUCs of the training and testing cohorts were 0.64 ± 0.03/0.62 ± 0.07, 0.70 ± 0.03/0.67 ± 0.06, and 0.75 ± 0.03/0.71 ± 0.07, respectively, and for 2%/2 mm, the average AUCs of the training and testing cohorts were 0.72 ± 0.03/0.72 ± 0.06, 0.78 ± 0.04/0.73 ± 0.07, and 0.81 ± 0.03/0.75 ± 0.06, respectively. In the classification, the PF model has a better classification performance than the CF model. Moreover, the HF model provides the best result among the three classifications models.

**Conclusions:** The planomics features can be used for predicting and classifying the GPR results and for improving the model performance after combining the conventional features for the GPR classification.

## Introduction

Volumetric modulated arc therapy (VMAT) is advanced radiotherapy (RT) technology commonly adopted into the clinic. As a standard procedure, the patient-specific quality assurance (QA) will be performed prior to the RT delivery to evaluate the quality of the RT plan, which is recommended by the report of the American Association of Physicists in Medicine Task Group (TG) Nos. 119 and 218 (Ezzell et al., 2009; Miften et al., 2018). In the evaluation, a gamma passing rate (GPR) is derived from the measured and calculated dose of the treatment planning system (TPS). Under the recommended gamma action limit (Ezzell et al., 2009), the plan is determined as “pass” or “fail.” However, the implementation of QA is time-consuming and adds a heavy clinical workload to the physicist.

With the development of technology, machine learning (ML) is widely applied in medical research (Jiang et al., 2021; Ma et al., 2021; Xia et al., 2021; Zhang et al., 2021a,b). To enhance the efficiency of QA, instead of the measured-based method, the ML technique has been involved in predicting the GPR by using the features of intensity-modulated radiotherapy (IMRT) or VMAT (Chiavassa et al., 2019; Chan et al., 2020; Kalet et al., 2020; Tomori et al., 2021). For example, Valdes et al. (2016) adopted the regression algorithm LASSO to predict the GPR of IMRT plans based on 78 aperture-based complexity metrics. In the following year, they investigated the prediction of GPR with different measurement techniques and across multiple institutions using the same algorithm (Valdes et al., 2017). All results showed that the ML technique is an efficient tool to predict the GPR. In addition to the prediction, Li et al. (2019) performed a multiple institutions analysis on the classification and prediction of GPR accounting for the VMAT plan by using ML algorithms and 54 complexity metrics. Ono et al. (2019) involved 28 complexity metrics to predict the VMAT GPR by building two models using multiple regression analysis and neural networks. All the above studies predict the plan GPR based on the complexity metric-based/aperture-based features (Du et al., 2014). The complexity metric is calculated by adopting the multiple leaf collimator (MLC) position, jaw position, gantry angle, and monitor units (MU) stored in the RT plan. A series of metrics (McNiven et al., 2010; Younge et al., 2012; Masi et al., 2013; Du et al., 2014; Park et al., 2014, 2015; Crowe et al., 2015; Götstedt et al., 2015; Sumida et al., 2017; Lam et al., 2019) have been developed to characterize the plan complexity correlated with the measured GPR, and those results demonstrate a good correlation with the measured GPR. Moreover, following the suggestion from Du et al. (2014), however, all complexity metrics attempt to characterize the treatment plan accuracy using a single complexity score, but they do not adequately distinguish plan heterogeneity.

Along with the plan complexity, another modeling approach, known as one of the fluence map complexity-based approaches, was developed. Nauta et al. (2011) analyzed the fluence map complexity employing fractal dimensions analysis. Interian et al. (2018) predicted the GPR of the IMRT plan using fluence maps-based features. Park et al. (2019) performed the texture analysis method on fluence maps of the VMAT plan. The fluence map for a VMAT plan is generated by superposing each fluence map of the control point. Tomori et al. (2018) adapted the dummy plans to train a model by using the two planar dose distributions. This method can make the modeling more efficient. To improve the model performance, Hirashima et al. (2020) combined complexity features and dosiomics features to predict and classify the GPR. However, the fluence-based approach has a limitation in that some fluence maps can be produced either by a single large beam or by a combination of successive small beams (Du et al., 2014).

The features of either the aperture-based approach or fluence map-based approach only consider the overall information of the treatment plan or dose distribution. For example, the complexity metrics of the modulation complexity score (MCS), small aperture score (SAS), plan area (PA), plan irregularity (PI), etc. are all overall characteristic metrics. Even with plenty of features extracted from the fluence map-based approach, the fluence map is generated from a superposition map. To overcome the limitation of using an overall feature, more attention should be paid to the information from each control point (Shiba et al., 2020). Inspired from the fluence map-based method (Park et al., 2019), the aperture-based metrics at each control point can be calculated based on the RT plan DICOM file, and then a series of statistical histogram data using metrics of all control points, noted as planomics, can be extracted as plan features.

Overall, this study will: (1) propose a new feature extraction method by considering complexity metrics at each control point and thereby (2) investigate the performance of new feature-based prediction and classification models with ML by using the head and neck (H&N) VMAT plan of the patient; (3) additionally, the combination of the two features will also be used to predict the GPR.

## Materials and Methods

### Data Characteristics

A total of 131 H&N plans in our institution from 2019 to 2021 were retrospectively collected. All plans adapted the 6 MV X-ray with VMAT technique by using 2–7 arcs, which were designed in the Eclipse^TM^ treatment planning system (V15.6, Varian Medical Systems, Palo Alto, CA, United States) using the medical linac of TrueBeam, VitalBeam, and Halcyon (Varian Medical Systems). These three models of medical linac are equipped with an electronic portal imaging device (AS1000). For TrueBeam and VitalBeam, 120 pairs of the MLC, whose maximum field size is 40 × 40 cm with 5 mm leaf width for central 20 cm of field and 10 mm for outer 20 cm of field. The Halcyon has a dual-layer MLC system with 57 pairs of MLC forming a maximum field size of 28 × 28 cm and 5 mm leaf width. In plan designing, a CT image with 3 mm slice spacing and grid spacing of the dose calculation with 3 × 3 × 3 mm was used to calculate dose distribution using algorithms of AAA version 15.6 in Eclipse. Besides, the dose rates of the plan and the minimum leaf gap were set as 600 MU/min (TrueBeam and VitalBeam) or 800 MU/min (Halcyon) and 0.2 cm, respectively. Moreover, all plans were optimized using gantry angle sampling of 2.0341° between the control points for Eclipse.

### Dosimetric Verification

Dosimetric verification for all plans was carried out in the corresponding linac by using the portal dosimetry system (Version 15.6) integrated into Eclipse based on the EPID. A comparison between the measured dose and the TPS-calculated dose for all plans was performed using gamma analysis in terms of three gamma indices of 3%/3 mm, 3%/2 mm, and 2%/2 mm with a 10% dose threshold. Besides, the absolute dose model and global normalization were adapted to the gamma analysis.

### Conventional Features

Forty-eight conventional features affecting the dose delivery accuracy were used in the study, as shown in Table 1 (McNiven et al., 2010; Nauta et al., 2011; Younge et al., 2012; Masi et al., 2013; Du et al., 2014; Park et al., 2014, 2015; Crowe et al., 2015; Götstedt et al., 2015; Li et al., 2019). All features were calculated by an in-house-developed Python script based on the RT plan DICOM files extracted from the Eclipse TPS. The rest of the six features used in the paper (Li et al., 2019) were not included in our study owing to the non-consistent number of VMAT arcs used in the treatment plan. In addition, the angle range of the VMAT arc for some patient plans was also non-consistent. In this case, the Python script has been developed specifically to address those problems. Besides, the script has been updated to deal with the difference in dose rates and the number of MLC leaves.

**TABLE 1:** Conventional plan feature metrics.

Quantity	Metrics	References
12	Modulation index for leaf speed (MI_*s*_, *f* = [0.2, 0.5, 1, 2])	Park et al., 2014
	Modulation index for leaf acceleration (MI_*a*_, *f* = [0.2, 0.5, 1, 2])	
	Modulation index for total (MI_*t*_, *f* = [0.2, 0.5, 1, 2])	
13	The proportion of leaf speed ranging from *a*_*i*_ − *a*_*i*1_(*S*_*a*_*i*_ − *a*_*i*1__) with *a* = [0, 0.4, 0.8, 1.2, 1.6, 2.0] and *i* = 1, 2,…, 5.	Park et al., 2015
	The proportion of leaf acceleration ranging from *b*_*i*_ − *b*_*i*1_(*A*_*b*_*i*_ − *b*_*i*1__) with *b* = [0, 1, 2, 4, 6] and *i* = 1, 2, 3, 4.	
	Average leaf speed (ALS), standard deviation of leaf speed (SLS) Average leaf acceleration (ALA), standard deviation of leaf acceleration (SLA)	
4	Small aperture score (SAS, *l* = 5, 10, 20mm) Mean asymmetry distance (MAD)	Crowe et al., 2015
3	Modulation complex score (MCS), leaf sequence variability (LSV), aperture area variability (AAV)	McNiven et al., 2010
5	Plan area (PA), plan irregularity (PI), plan modulation (PM), plan normalized MU (PMU), union aperture area (UAA)	Du et al., 2014
1	Edge metric (EM)	Younge et al., 2012
3	Converted aperture metric (CAM), edge area metric (EAM), circumference/area (C/A)	Götstedt et al., 2015
2	Average leaf travel distance (ALT), combination of ALT and MCS (ALTMCS)	Masi et al., 2013
2	Average leaf gap (ALG), standard deviation of leaf gap (SLG)	Nauta et al., 2011
3	Average dose rate (ADR), standard deviation of dose rate (SDR), prescribed dose to primary target per fraction (dose)	Li et al., 2019

### Planomics Features

The planomics features were calculated using the DICOM-RT plan files. The planomics feature is a kind of control point-based complexity metric. The calculation and definition of the planomics features were integrated into Supplementary Section 1. All features were calculated using an in-house Python code.

### Machine Learning Model Construction and Validation

**FIGURE 1:**
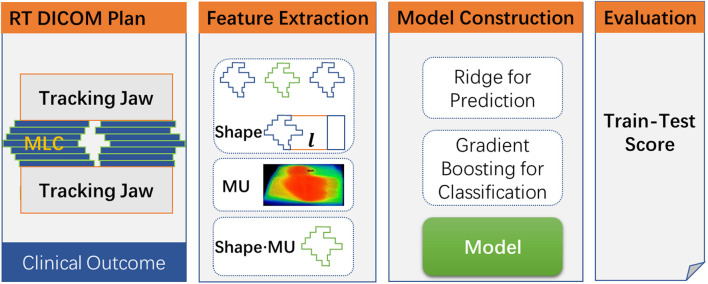
The overall workflow for the study.

The overall workflow of this study is shown in Figure 1. The DICOM plan data are firstly extracted from the TPS, and then conventional and planomics features were calculated based on the above approach. After combining with clinical outcome, two types of the model will be built, namely, the (a) prediction model and (b) classification model. Finally, the model was evaluated by using a multiple train–test score based on evaluation metrics. In the study, the models of regression prediction and classification are trained based on the Python package Scikit-learn (Pedregosa et al., 2011).

**FIGURE 2:**
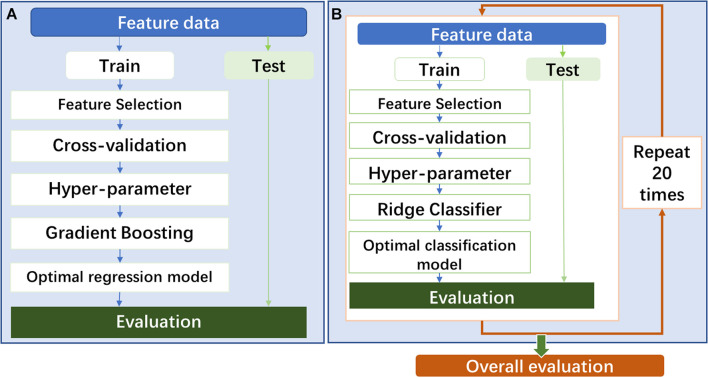
**(A)** The procedure of prediction model construction. **(B)** The procedure of classification model construction.

In the prediction model construction, the procedure of model construction is shown in Figure 2A. As shown in the figure, all feature data were divided into two parts: training (70%) and testing (30%) cohorts (train–test split). In the training cohort, the stable and predictive features were firstly screened out in the step of feature selection. Following that, a gradient boosting regressor (GBR) was adapted to construct the prediction model. To get an optimal regression model, two improved approaches were used: (1) random grid search for achieving optimal hyperparameter of GBR and (2) fivefold cross-validation for tuning parameters in the model.

**FIGURE 3:**
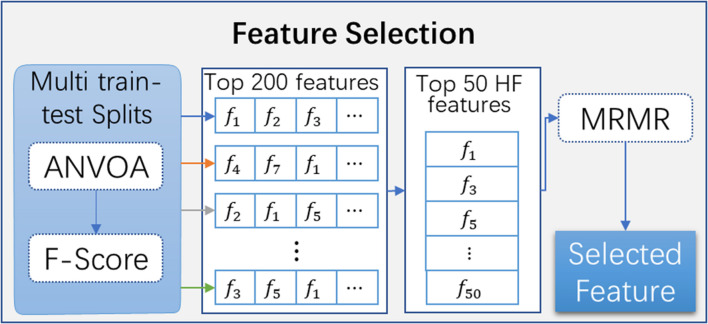
The method for feature selection. HF, high frequency; ANOVA, analysis of variance; MRMR, minimum redundancy maximum relevance.

In the feature selection (Figure 3), the features were: (1) firstly removed by using an unsupervised feature selection method of the analysis of variance (ANOVA) with zero thresholds, and then (2) a large number of features were selected based on the *F*-score by using the *F*-test-based univariate feature selection method. To obtain a set of features with stability and robustness, features were selected by repeating the above two steps for a hundred iterations. The *30* features with the highest frequency were screen out based on the number of occurrences in the 100 sets of selected features. Eventually, the *k* features were selected by using minimum redundancy maximum relevance (MRMR). It should be mentioned that the testing label in the 100 iterations is not involved in eliminating the effect of data leakage. Besides, the appropriated maximum number of features in a model should be smaller than 10% of the sample size (Abu-mostafa, 1995). Therefore, in the study, the *k* is set as *10* for building models.

In the classification model construction, the model procedure of model construction is shown in Figure 2. The model will be constructed by following almost the same procedure of the prediction model construction. One difference is that a classifier of linear least squares with l2 regularization (Ridge) was used to build the classification model. Another difference is that the whole modeling process will repeat 20 times to evaluate the overall predictability performance of the classification model using several different feature modalities. Based on the TG 218 report (Miften et al., 2018), the action limit was used to act VMAT plan as “pass” or “fail.” In the study, stricter action limits were chosen with 99% for 3%/3 mm, 98% for 3%/2 mm, and 95% for 2%/2 mm.

According to the types of feature modality, three kinds of the model were constructed separately using the training cohort for the prediction and classification models, respectively: (1) using conventional features (CF), (2) using planomics features (PF), and (3) using hybrid features (HF). After feature modeling, a test cohort was used to assess the performance of two kinds of models by using one metric of the absolute prediction error (APE) using *APE* = |*GPR*_*pred*_ − *GPR*_*meas*_|/*GPR*_*meas*_ for the prediction model and one metric of the area under the receiver operating characteristic (ROC) curve (AUC) for the classification model.

## Results

### Measured Gamma Passing Rates

Between 2019 and 2021, 131 valid patient plan data were involved in the study after data cleaning based on plan data integrity. The distributions of GPR for total, training, and test cohorts are listed in Table 2. As shown in Table 2, the majority of GPR was distributed into 95–100% for 3%/3 mm, 90–100% for 3%/2 mm, and 85–98% for 2%/2 mm. Based on the distributions, the strict gamma threshold and ratios of the corresponding samples were 99% and 53.9%/46.1% for 3%/3 mm, 98% and 48.4%/51.6% for 3%/2 mm, and 95% and 40.0%/60.0% for 2%/2 mm.

**TABLE 2:** The summary information of different gamma criteria.

Gamma indices	3%/3 mm	3%/2 mm	2%/2 mm
	Total, *N* (%)	Total, *N* (%)	Total, *N* (%)
[99%, 1]	86 (57.3)	51 (34.0)	22 (14.7)
[98%, 99%]	28 (18.7)	27 (18.0)	10 (6.7)
[95%, 98%]	33 (22.0)	43 (28.7)	35 (23.3)
[90%, 95%]	3 (2.0)	24 (16.0)	56 (37.3)
[85%, 90%]	0 (0)	5 (3.3)	18 (12.0)
[70%, 85%]	0 (0)	0 (0)	9 (6.0)
Mean ± SD	[98.7 ± 1.5]%	[97.3 ± 2.7]%	[93.8 ± 5.0]%

### Feature Information

In the feature extraction, a total of 48 conventional features and 2,476 planomics features for each VMAT plan were calculated by analyzing the plan DICOM files using the package of Pydicom 2.1.2 based on Python 3.7 (Mason, 2011). After feature selection, the used features in the prediction and classification models are collected in Supplementary Tables 4, 5.

### Model Performance

The hyperparameters in the three regression models using the hybrid features are all the same with alpha of 0.12, learning rate of 0.12, number of estimators of 58, and maximum depth of 3. In the classification model, 30 classification models using hybrid features for each GPR were constructed, and the hyperparameter in the Ridge classifier was different model to model with the two most frequent alpha values of 31.6 and 1.0.

#### Prediction Accuracy

**FIGURE 4:**
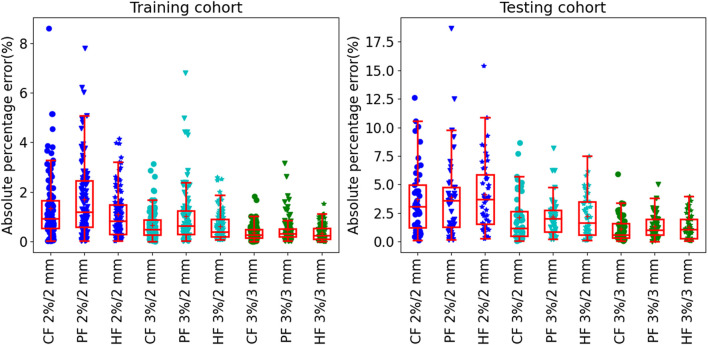
The absolute prediction error by using different feature modalities at gamma criteria of 2%/2 mm, 3%/2 mm, and 3%/3 mm for the training **(left figure)** and testing **(right figure)** cohort, respectively. All solid circles, triangles, and pentagrams are the APE values. The red box shows the quartile values of each set of APE data. The red solid line and red plus inside the box are the median and average values, respectively.

The results of prediction accuracy are shown in Figure 4. At gamma criteria of 2%/2 mm, the average APE (red plus) by using CF, PF, and HF was 1.3 ± 1.2%, 1.7 ± 1.5%, and 1.1 ± 1.0% in the training cohort and 3.6 ± 3.0%, 3.8±3.5%, and 4.1 ± 3.1% in the testing cohort, respectively. At gamma criteria of 3%/2 mm, the average APE (red plus) by using CF, PF, and HF was 0.7 ± 0.6%, 1.0±1.1%, and 0.6 ± 0.6% in the training cohort and 2.0 ± 2.0%, 2.2 ± 1.8%, and 2.2 ± 1.9% in the testing cohort, respectively. At gamma criteria of 3%/3 mm, the average APE (red plus) for using CF, PF, and HF was 0.4 ± 0.3%, 0.4±0.5%, and 0.3±0.3% in the training cohort and 1.2 ± 1.2%, 1.3 ± 1.0%, and 1.2 ± 1.1% in the testing cohort, respectively. All average APEs were smaller than 2.0 and 5.0% for the training and testing cohorts, respectively.

Table 3 shows the summary of the average prediction error at three gamma criteria of 3%/3 mm, 3%/2 mm, and 2%/2 mm. There are no statistically significant prediction errors among each three prediction models at the three gamma criteria. More than 90% of the plans can be predicted precisely with lower than 3.0% APE for 3%/3 mm, 5.0% APE for 3%/2 mm, and 10% for 2%/2 mm. The MAPE of the three prediction models was smaller than 1.3% for 3%/3 mm, 2.2% for 3%/2 mm, and 4.1% for 2%/2 mm.

**TABLE 3:** The summary average prediction error in the testing cohort by using the three feature modalities at different gamma criteria.

Metrics		APE < 3.0%	APE < 5%	APE < 10%	MAPE (SD%)
3%/3 mm	CF, *n* (%)	42 (93.3)	44 (97.8)	45 (100)	1.2% (*1*.2)
	PF, *n* (%)	43 (95.6)	44 (97.8)	45 (100)	1.3% (1.0)
	HF, *n* (%)	41 (91.1)	45 (100)	45 (100)	1.2%(1.1)
3%/2 mm	CF, *n* (%)	35 (77.8)	42 (93.3)	45 (100)	2.0%(2.0)
	PF, *n* (%)	36 (80.0)	42 (93.3)	45 100)	2.1% (*1*.7)
	HF, *n* (%)	29 (64.4)	41 (91.1)	45 (100)	2.2% (*1*.8)
2%/2 mm	CF, *n* (%)	23 (51.1)	35 (77.8)	43 (95.6)	3.6% (3.1)
	PF, *n* (%)	19 (42.2)	35 (77.8)	43 (95.6)	3.8% (3.5)
	HF, *n* (%)	20 (44.4)	30 (66.7)	43 (95.6)	4.1% (3.2)

*MAPE, mean absolute prediction error, SD, standard deviation; CF, conventional features; PF, planomics features; HF, hybrid features.*

In all the three hybrid prediction models, the final selected features are the same, that is, *MCS*, *S*_*0–0.4*_, *S*_*0.4–0.8*_, *MI*_*a* 2−4_, *MI*_*a* 4−6_, *ALT*, *AAV*, *ALTMCS*, *MUAP*_*TR* 24,200−24,400_, and *MUAP*_*T 24,200–24,400*_. Eight features are from the conventional feature and the other two features are from the planomics feature.

#### Classification Accuracy

**FIGURE 5:**
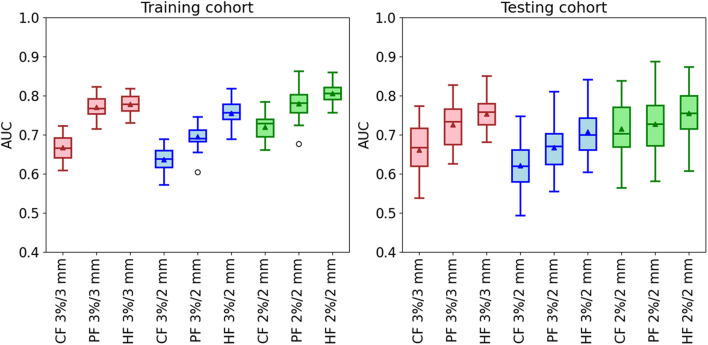
The AUC of the classification model in the training and testing cohorts by using three kinds of feature modalities in the conditions of the three gamma criteria. The used action limit is 99% for 3%/3 mm, 98% for 3%/2 mm, and 95% for 2%/2 mm.

The classification results of the model with rigorous action limit (99, 98, and 95% for 3%/3 mm, 3%/2 mm, and 2%/2 mm, respectively) are shown in Figure 5. For the gamma criteria of 3%/3 mm, the average and SD AUCs of the training and testing cohorts were 0.67 ± 0.03/0.66 ± 0.07, 0.77 ± 0.03/0.73 ± 0.06, and 0.78 ± 0.02/0.75 ± 0.04, respectively. For the gamma criteria of 3%/2 mm, the average AUCs of the training and testing cohorts were 0.64 ± 0.03/0.62 ± 0.07, 0.70 ± 0.03/0.67 ± 0.06, and 0.75 ± 0.03/0.71 ± 0.07, respectively. For the gamma criteria of 2%/2 mm, the average AUCs of the training and testing cohorts were 0.72 ± 0.03/0.72 ± 0.06, 0.78 ± 0.04/0.73 ± 0.07, and 0.81 ± 0.03/0.75 ± 0.06, respectively.

From Figure 5, it is clear that the three hybrid classification models have the best performance in all the three gamma criteria. It is improved by 16 and 14% for the training and testing cohorts at 3%/3 mm, 17% and 11% for the training and testing cohorts at 3%/2 mm, and 13% and 4% for the training and testing cohorts at 2%/2 mm. Besides, the classification model using the planomics feature has better predictability compared with the conventional model. The final selected features in the three hybrid models are shown in Supplementary Table 1. Most of the features used in the hybrid model are planomics features, especially at 3%/2 mm with all planomics features.

## Discussion

In this study, we proposed a novelty feature, namely, the planomics feature, to comprehensively characterize the quality of the radiotherapy plan with regression and classification analysis by using machine learning techniques. To the best of our knowledge, the features from each control point are firstly involved in the analysis of GPR. Only a similar study of Park et al. (2015) counted the MLC leaf speed and accelerations with a limit of five ranges for the VMAT plan, which had a limitation on the description of plan complexity. The other studies just adapted overall values calculated from each control point. For example, McNiven et al. (2010) adapted the summation to calculate MCS, AAV, and LSV. In the study of Du et al. (2014), they proposed features (i.e., aperture area, aperture shape irregularity, and degree of beam modulation) that were calculated by the summation of each control point value. Likewise, Younge et al. (2012) applied the edge metric which was a summation using edge length and the aperture at each control point. In addition, Götstedt et al. (2015) utilized an average value of three metrics (convert edge metric, edge area metric, and the ratio *circumference/area*) at each point. The other complexity metrics in Table 1 are calculated based on the methods of summation, average, or standard deviation.

For the model performance, the regression results show that the APE has consistent results for all prediction models at the three gamma criteria, which agreed with the result of the study of Li et al. (2019) that more than 90% of plans had APE <3.5% for 2%/2 mm and 5% for 3%/2 mm. In the classification, the results demonstrated well that our proposed planomics features can distinguish heterogeneity in terms of the H&N VMAT plan more efficiently compared with the conventional features. Besides, hybrid models for three GPR thresholds achieve the best performance with the AUC increment of 4–16% in the testing cohort compared with the conventional and planomics models.

In the three hybrid regression models, only two MUAP planomics features were selected. It shows that the conventional features have stronger correlations with the measured GPR. In contrast, for the hybrid classification model, the selected features are dominated by the planomics features. It means that the planomics have a better relevance with GPR results of “pass” or “fail.” Besides, for the selected features (see Supplementary Table 5), most of the planomics features are related to MU or MUAP with seven, eight, and four features for 3%/3 mm, 3%/2 mm, and 2%/2 mm, respectively. It indicates that more attention should be paid to the MU in the planning design. One PSR feature, similar to the BI metric (Du et al., 2014), is contained in all three hybrid classification models. It shows a good correlation with the GPR results for different gamma criteria. In addition, SF features are included in the hybrid classification model of 3%/3 mm and 3%/2 mm. Only two RF features and one area feature are used in the classification model at 2%/2 mm, which have a weaker connection with GRP results.

Based on the recommendation of the TG 218 report (Ezzell et al., 2009), the action limit of 3%/2 mm is 90% with a 10% dose threshold. In our study, stricter action limits were used with 98% at 3%/2 mm with a 10% dose threshold. As mentioned by Li et al. (2019), to achieve an adequate amount of low GPR plans for modeling is too difficult to build a balanced dataset in a single institution. The imbalanced dataset is common in medical diagnostics, text classification, face, and image recognition, and also in GPR studies (Valdes et al., 2016; Tomori et al., 2018; Li et al., 2019). The imbalanced data can affect classifiers to weaken the performance of the classification model, as most classifier learning algorithms are accountable for a relatively balanced distribution (Sun et al., 2009; Kaur et al., 2019; Thabtah et al., 2020). Solutions on the imbalance are to obtain more data or adopt a specific algorithm (Kaur et al., 2019). To avoid the effect from the imbalanced data, a straightforward way is to set stricter action limits to obtain a relatively balanced class data distribution. Based on the data distribution, the stricter action limits of 99, 98, and 95% were chosen for 3%/3 mm, 3%/2 mm, and 2%/2 mm, respectively. In addition, another set of action limit with 98, 95, and 90% was chosen to evaluate the classification model performance in all gamma criteria. The modeling results using multiple train–test splits are shown in Supplementary Figure 3. The data distribution with the label “pass” and “fail” was 76.0%/24.0% at 3%/3 mm, 80.7%/19.3% at 3%/2 mm, and 82.0%/18.0% at 2%/2 mm. In the modeling, a downsampling method was used to reduce the effect of imbalanced data. The figure presents the same results with Figure 5, where our proposed planomics features have better predictability and the hybrid models give the best results, too.

Instead of a single train–test split, an approach with multiple train–test splits was used to evaluate the predictability of three sets of features to classify the GPR result. One main concern is that a single train–test split generates a random feature data distribution in training and testing cohorts. In some cases, one set of features has a stronger correlation in two cohorts, yet another set of features has a weaker correlation in two cohorts. In other cases, the result may give an opposite result. To avoid this randomness, the approach with multiple train–test splits was involved in the study.

The shortcoming of the study is that only H&N VMAT plans were involved, and data for just only one institution were included in the study. For the VMAT plans of the other site, the IMRT plans, and data from multiple institutions, the performance of the planomics should be further investigated. Besides, the other complexity metrics will also be evaluated in a future work.

## Conclusion

Our proposed control point-based planomics feature can be used to predict and classify the measured GPR for patient-specific quality assurance. In the regression prediction, the planomics and conventional features give a similar modeling performance. In the classification, the predictability of the planomics feature is better than the conventional aperture-based complexity metric. Besides, the combination using planomics and conventional features provides the best result in the classification.

## Data Availability Statement

The datasets generated for this study are available on request to the corresponding author.

## Ethics Statement

The study was reviewed and approved by the Hospital Ethical Committee of the Affiliated Cancer Hospital of Zhengzhou University.

## Author Contributions

BL and JC equally contribute to the study for data analyzing and writing. HG designed and organized the study. WG, RM, and HL collected the image data. ZL, XC, TC, and DL carried out the dose measurement of patient QA. XZ, TW, and HT collected the clinical information. All authors contributed to the article and approved the submitted version.

## Conflict of Interest

The authors declare that the research was conducted in the absence of any commercial or financial relationships that could be construed as a potential conflict of interest.

## Publisher’s Note

All claims expressed in this article are solely those of the authors and do not necessarily represent those of their affiliated organizations, or those of the publisher, the editors and the reviewers. Any product that may be evaluated in this article, or claim that may be made by its manufacturer, is not guaranteed or endorsed by the publisher.
