# Supplementary material for: Improvement Using Planomics Features on Prediction and Classification of Patient-Specific Quality Assurance Using Head and Neck Volumetric Modulated Arc Therapy Plan
Source: Front Neurosci. 2021 Oct 1;15:744296. doi: 10.3389/fnins.2021.744296 (PMC8517188; doi:10.3389/fnins.2021.744296)
Supplement: Supplementary file 1 [file Data_Sheet_1.docx]

Supplementary Material

# Calculation of planomics feature

The planomics feature is calculated based on two kinds of parameters, such as shape factors and the monitor's units. The definition of the two parameters is as follows. After that, the calculation of the planomics feature is given in Subsection 1.3.

## Shape factors

The shape factors consist of the four kinds of parameters, such as area of sub-field, perimeter to surface ratio, rectangular factor, and square factor. The definition of each factor is as follows.

- Area of sub-field
  A treatment VMAT plan for radiotherapy generated from TPS is composed of a series of control points. For each control point, there are two components, such as sub-field and monitor unit. The sub-field with various shapes was formed by 120 MLC and a tracking jaw, and one example is demonstrated in Supplementary Figure 1. As shown in Supplementary Figure 1 (b3), the area of the sub-field is defined as the area of the enclosed area surrounding by MLC and jaws.
- Perimeter to the surface ratio (PSR)
  As shown in Supplementary Figure 1, the perimeter is the side length of the polygonal sub-field. Hence, the perimeter to surface ratio is defined as:

$$\boldsymbol{PSR=}\frac{\boldsymbol{P}}{\boldsymbol{S}} ,$$

where $P$ and $S$ represent the perimeter and the area of the subfield, respectively.
- Rectangular factor (RF)
  As shown in Supplementary Figure 1 (b5), an equivalent rectangle with the same area to the sub-field is generated based on the area $S$ and the length $l$ of the sub-field. The width of the equivalent rectangle can be expressed as $d=S/l$. The rectangular factor is defined as:

$$\boldsymbol{RF=}\frac{\mathbf{min}\boldsymbol{(l, d)}}{\mathbf{max}\boldsymbol{(l, d)}} ,$$

where $min(*)$ and $max(*)$ are the operator to get the minimum and maximum respectively. From the equation, the value of $RF$ is always smaller than or equal to 1 and larger than 0, i.e., $0<RF\leq1$.
- Square factor (SF)

Based on the previous factor, the square factor is defined as:

$$\boldsymbol{SF}=\frac{\boldsymbol{P}^{\boldsymbol{2}}}{\boldsymbol{8\times S}}.$$

From the above equation, the range of $SF$ is larger than or equal to 1, and the value of $SF$ is 1 when and only when the perimeter of sub-field is equal to the perimeter of the equivalent rectangle and at the same time the rectangle is square, i.e.,

$$\boldsymbol{SF=}\left\{ \begin{aligned} \begin{matrix} \boldsymbol{1,} & \boldsymbol{P=2}\left( \boldsymbol{l+d} \right)\boldsymbol{, l=d} \end{matrix} \\ \begin{matrix} \boldsymbol{>1,} & \boldsymbol{other conditions} \end{matrix} \end{aligned} \right..$$

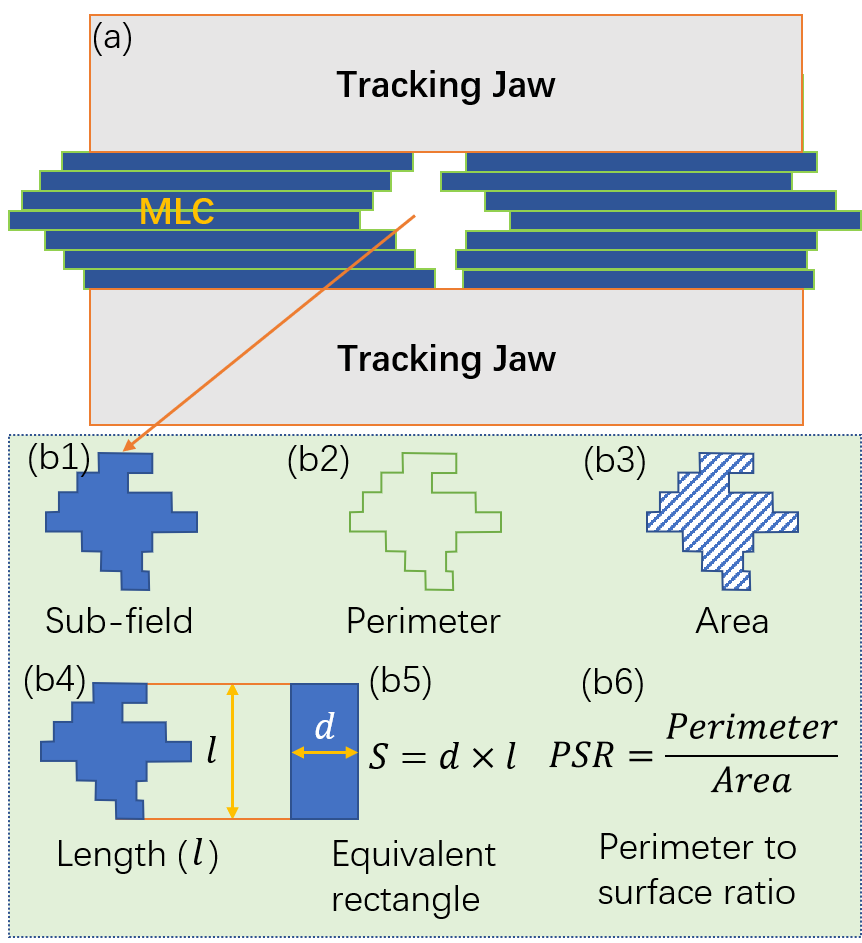


Supplementary Figure 1 (a) Demonstration for the sub-field surrounding by MLC and two jaws. (b) Demonstration of the shape factors. (b1) Polygonal sub-field; (b2) Perimeter of sub-field; (b3)Area of the sub-field; (b4) Sub-field length; (b5) Equivalent rectangle with its width; (b6) Perimeter to surface ratio.

## Monitor units (MU) of sub-field & MU area product (MUAP)

In a control point, another component is the monitor unit (MU), which is related to the delivery dose from linac to the patient. For each sub-field, a corresponding MU was also given by the DICOM plan file. The value of MU is optimized and calculated in the treatment planning system, and is typically large than or equal to 0 (i.e., $\mathrm{MU}\geq0$).

MU area product (MUAP) is the product of MU and area of the corresponding sub-field:

$$MUAP=MU\times S,$$

which is larger than or equal to 0, i.e., $MUAP\geq0$.

## Planomics feature value

For a VMAT plan, a large number of control points were used. Here, an example is given to demonstrate the planomics feature. For example, with a VMAT plan with one 360° arc, a total of 180 control points was generated with a step of 2°. Correspondingly, there are 180 values in terms of each above modality. Based on the 180 values, noted as $X=\left[ x_{1},x_{2},\ldots, x_{i}, \ldots,x_{180} \right]$, two kinds of features called first-order and histogram information are extracted as the planomics feature. a) As shown in Supplementary Table 2, there are six types of first-order features, including minimum, maximum, standard deviation (STD), the average value (mean), summation of all values (sum), and median value. b) A series of intervals or bins, $\left[ a_{0},a_{1},a_{2},\ldots, a_{i}, \ldots, a_{m} \right]$ (the last element $a_{m}$ is set as infinity, i.e., $a_{m+1}=\infty$), is firstly given to get the histogram information. Then, four kinds of features were defined as (1) quantity values $Q=[Q_{1}, Q_{2}, \ldots, Q_{i}, \ldots, Q_{m}]$ , where$Q_{i}$ is the number of values greater than $a_{i}$ and less than $a_{i+1}$; (2) summation values $[T_{1}, T_{2}, \ldots, T_{i}, \ldots, T_{m}]$, where $T_{i}$ is the summation of values greater than $a_{i}$ and less than $a_{i+1}$; (3) quantity ratios $\left[ Q_{1}, Q_{2}, \ldots, Q_{i}, \ldots, Q_{m} \right]/\sum_{1}^{\infty} Q_{i}$; (4) summation ratios $\left[ T_{1}, T_{2}, \ldots, T_{i}, \ldots, T_{m} \right]/\sum_{1}^{\infty} T_{i}$ (see Supplementary Table 2).

For each feature modality, related bins should be determined appropriately based on two considerations that are feature quantity and validity of data distribution. The feature quantity will be a large amount for a small bin width, which will increase the complexity in analyzing. On the other hand, the data distribution cannot be characterized accurately with a large bin width, which leads to a poor heterogeneity of data. Taking account of the two considerations, six kinds of bins were selected by using all patient plan data, as shown in Supplementary Table 3. Accordingly, the histogram information of all plan data for each modality is shown in Supplementary Figure 2. An in-house-developed Python script was used to extract features by analyzing the MLC positions and MU weights of all control points in VMAT plans.

Supplementary Table 2 Demonstration for the feature type.

| Type | Name or Definition |
| --- | --- |
| First-order | Minimum, Maximum, STD, Mean, Sum, Median |
| Histogram information  ($\left[ \boldsymbol{a}_{\boldsymbol{1}}\boldsymbol{,}\boldsymbol{a}_{\boldsymbol{2}}\boldsymbol{, \ldots,}\boldsymbol{a}_{\boldsymbol{i}}\boldsymbol{, \ldots,}\boldsymbol{a}_{\boldsymbol{i}} \right]$ is a bin width, where $\boldsymbol{i=1, 2, \ldots, m}$.) | $Q_{i}=\mathrm{Card}\left\{ (x_{i})\left\vert\left( x_{i}\in X,a_{i}\leq X <a_{i+1}, j=1,2,\ldots,180, i=1,2,\ldots,m \right) \right. \right\}$ |
|  | $T_{i}=\sum\left\{ (x_{i})\left\vert\left( x_{i}\in X,a_{i}\leq X <a_{i+1}, j=1,2,\ldots,180, i=1,2,\ldots,m \right) \right. \right\}$ |
|  | ${QR}_{i}=\frac{Q_{i}}{\sum_{1}^{\infty} Q_{i}}, with \sum{QR}_{i}=1, i=1,2,\ldots, m$ |
|  | ${TR}_{i}=\frac{T_{i}}{\sum_{1}^{\infty} T_{i}}, with\sum{TR}_{i}=1 , i=1,2,\ldots, m$ |

Supplementary Table 3 The bins of histogram information for each modality.

| Modality | Bins |
| --- | --- |
| Area | $\boldsymbol{a}_{\boldsymbol{i}}\boldsymbol{=}\left\{ \begin{aligned} \boldsymbol{200\times i, i=1,\ldots, 100} \\ \boldsymbol{\infty, i=101} \end{aligned} \right.$ |
| PSR | $\boldsymbol{a}_{\boldsymbol{i}}\boldsymbol{=}\left\{ \begin{aligned} \boldsymbol{0.01\times i, i=1,\ldots, 80} \\ \boldsymbol{\infty, i=81} \end{aligned} \right.$ |
| RF | $\boldsymbol{a}_{\boldsymbol{i}}\boldsymbol{=}\left\{ \begin{aligned} \boldsymbol{0.008\times i, i=1,\ldots, 100} \\ \boldsymbol{\infty, i=101} \end{aligned} \right.$ |
| SF | $\boldsymbol{a}_{\boldsymbol{i}}\boldsymbol{=}\left\{ \begin{aligned} \boldsymbol{0.5\times i, i=1,\ldots, 80} \\ \boldsymbol{\infty, i=81} \end{aligned} \right.$ |
| MU | $\boldsymbol{a}_{\boldsymbol{i}}\boldsymbol{=}\left\{ \begin{aligned} \boldsymbol{0.05\times i, i=1,\ldots, 100} \\ \boldsymbol{\infty, i=101} \end{aligned} \right.$ |
| MUAP | $\boldsymbol{a}_{\boldsymbol{i}}\boldsymbol{=}\left\{ \begin{aligned} \boldsymbol{200\times i, i=1,\ldots, 150} \\ \boldsymbol{\infty, i=151} \end{aligned} \right.$ |


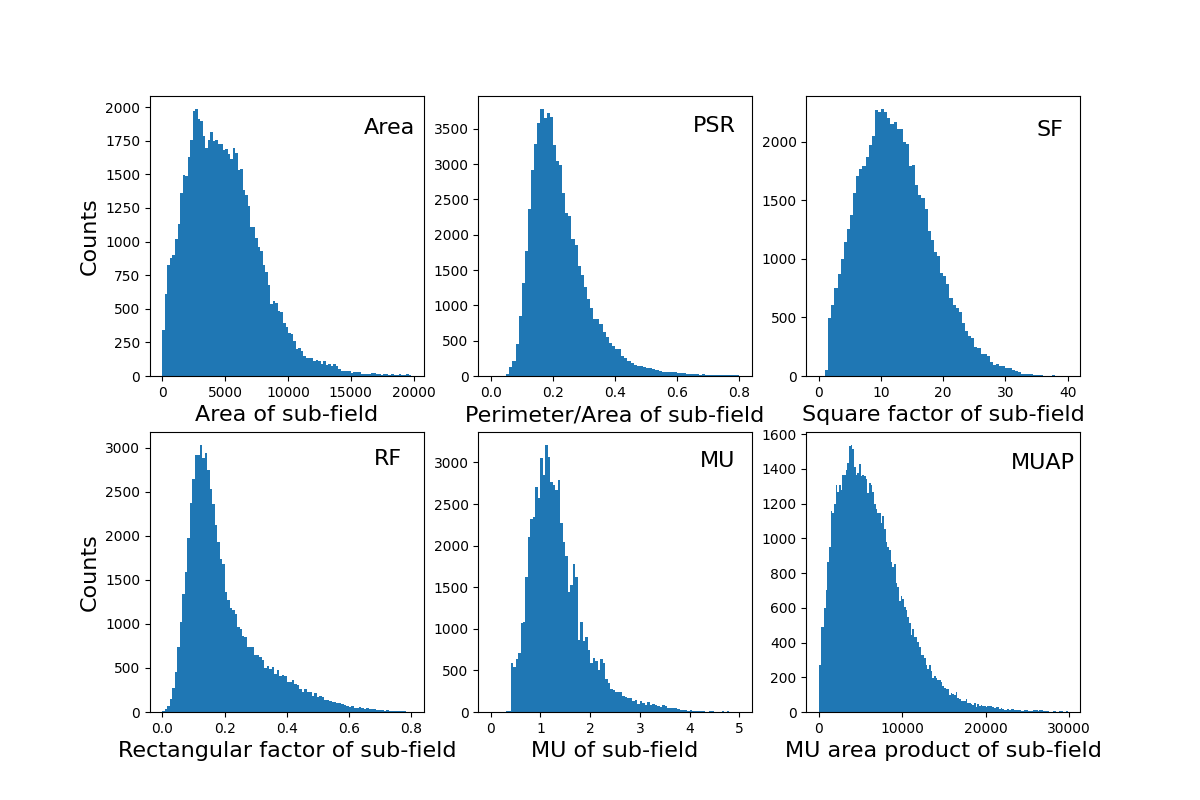


Figure 2 The histogram information of six modalities using the bins in Table 2.

## Supplementary Tables

Supplementary Table 4 The selected features of the three-hybrid regression prediction model at the gamma criteria of 3%/3 mm, 3%/2 mm, 2%/2 mm.

| Gamma criteria | Conventional feature | Planomics feature |
| --- | --- | --- |
| 3%/3 mm | S_0.4-0.8_, A_4-6_, A_2-4_, ALT, S_0-0.4_, MCS, AAV, ALTMCS | Area_Subdose_SUMRates_Betweeen24200_24400, Area_Subdose_SUM_Between24200_24400, |
| 3%/2 mm | A_2-4_, S_0.4-0.8_, A_4-6_, AAV, ALT, S_0-0.4_, MCS | Perimeter_Area_SUM_Between0.69_0.70, Perimeter_Area_Num_Between_0.690_0.70, Perimeter_Area_SUM_Between0.51_0.52 |
| 2%/2 mm | A_2-4_, S_0.4-0.8_, A_4-6_, ALT, AAV, MCS | Square_SUM_Between38.5_39.0, Square_Num_Between_38.5_39.0, Perimeter_Area_SUM_Between0.73_0.74, Perimeter_Area_SUM_Between0.69_0.70 |

*MUAP: the product of MU and area, SF: shape feature, PSR: perimeter to the surface ratio, RF: rectangular factor, Q: quantity, T: summation, R: ratio.

Supplementary Table 5 The selected features of the three-hybrid classification model at the gamma criteria of 3%/3 mm, 3%/2 mm, 2%/2 mm.

| Gamma criteria | Conventional feature | Planomics feature |
| --- | --- | --- |
| 3%/3 mm | ADR | MUAP_TR_20400-20600, SF_QR_38.5_39.0, MUAP_TR_27200_27300, MUAP_TR_25200_25400, MU_Q_1.1-1.15, MU_QR_0.45-0.5, PSR_TR_0.61_0.62, MU_TR_1.65-1.7, MU_Q_2.35-2.4 |
| 3%/2 mm | N/A | MUAP_TR_7200_7300, SF_QR_33.0-33.5, SF_T_11.5-12.0, PSR_TR_0.34-0.35, MU_QR_2.2-2.25, MU_QR_0.45-0.5, MU_T_4.7-4.75, MU_TR_4.6_4.65, MU_T_3.05-3.1, SF_TR_6400-6600 |
| 2%/2 mm | ADR, ALTMCS | MU_Q_1.1-1.15, RF_TR_0.792-0.8, MU_QR_0.45-0.5, MUAP_Q_5800-6000, MUAP_TR_24200-24400, PSR_QR_0.17-0.18, RF_Q_0.184-0.192, Area_T_10000-10200 |

*MUAP: the product of MU and area, SF: shape feature, PSR: perimeter to the surface ratio, RF: rectangular factor, Q: quantity, T: summation, R: ratio.

## Supplementary Figures


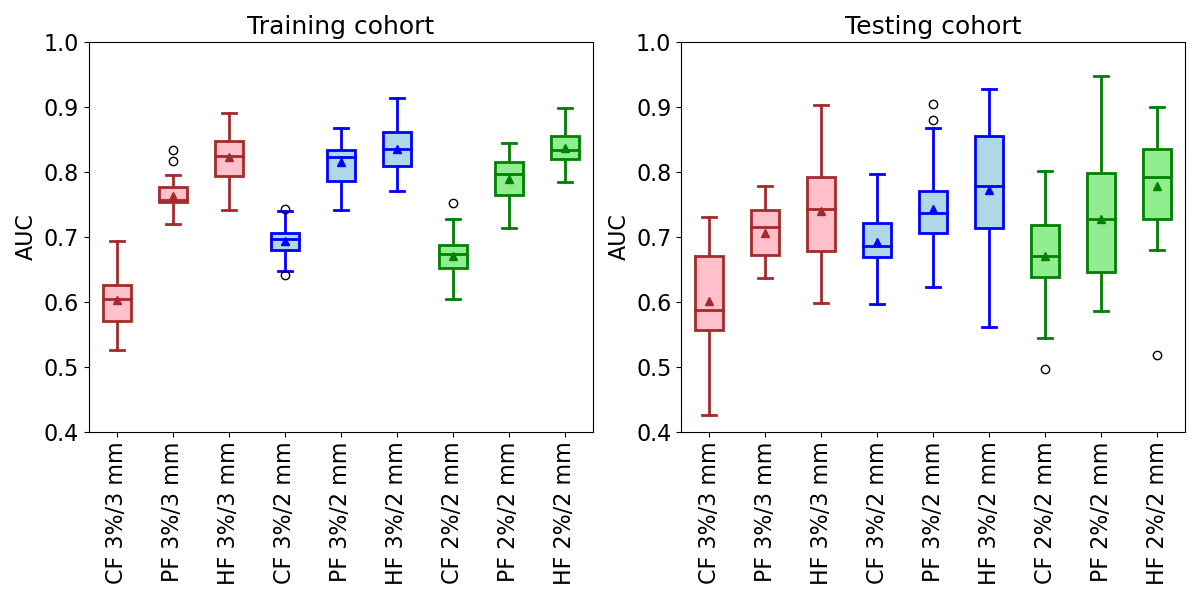


Supplementary Figure 3 The AUC of the classification model in the training and testing cohort by using three kinds of feature modalities in the conditions of the three gamma criteria. The used action limit is 98% for 3%/3 mm, 95% for 3%/2 mm and 90% for 2%/2 mm.
